# 11-oxygenated androgens in healthy young adults: Does use of hormonal contraceptives matter? The Fit Futures Study

**DOI:** 10.1007/s40618-025-02793-0

**Published:** 2026-02-06

**Authors:** Torkild Pettersen, Ole-Martin Fuskevåg, Yngve A. Figenschau, Elin K. Evensen, Anne-Sofie Furberg, Anne Winther, Guri Grimnes

**Affiliations:** 1 Tromsø Endocrine Research Group, Department of Clinical Medicine, UiT The Arctic University of Norway, Tromsø, Norway; 2 Department of Laboratory Medicine, University Hospital of North Norway, Tromsø, Norway; 3 Diagnostic Clinic, University Hospital of North Norway, Tromsø, Norway; 4 Department of Health and Care Sciences, UiT The Arctic University of Norway, Tromsø, Norway; 5 Department of Microbiology and Infection Control, University Hospital of North Norway, Tromsø, Norway; 6 Faculty of Health and Social Sciences, Molde University College, Molde, Norway; 7 Division of Internal Medicine, University Hospital of North Norway, Tromsø, Norway; 8 Division of Medicine, University Hospital of North Norway, Tromsø, Norway

**Keywords:** 11-oxygenated androgens, reference interval, combined hormonal contraceptives, population-based study, young healthy adults

## Abstract

**Purpose:**

Increasing evidence suggests a role of 11-oxygenated adrenal androgens (11-OxyA) (11-ketotestosterone, 11KT; 11β-hydroxytestosterone, 11OHT; 11-ketoandrostenedione, 11KA4; 11β-hydroxyandrostenedione, 11OHA4) in several hyperandrogenic disorders. This study aimed to describe distributions of 11-OxyA in a healthy young adult population and explore the relation between hormonal contraceptives and 11-OxyA.

**Methods:**

This study utilized cross-sectional data from the third Fit Futures Study conducted in Norway. 11-OxyA in fasting blood samples were analyzed by liquid chromatography tandem mass spectrometry technique (LC-MS/MS). Contraceptive use was registered and categorized as combined hormonal contraceptives (CHC) or gestagen-only contraceptives. Descriptive statistics were used to report 11-OxyA distributions. Independent t-tests and ANOVA were used to compare biomarker concentrations between groups.

**Results:**

The study included 289 males and 337 females with median age of 27 years. Males had 9–30% higher 11-OxyA concentrations than females (all p’s < 0.01). Among the females, 25.5% used CHC, 36.7% used gestagen-only contraceptives, and 37.8% used non-hormonal contraceptives or no contraceptives. As concentrations of 11-OxyA in gestagen-only contraceptives users were similar to non-users, these groups were combined. CHC users had 25–29% lower concentrations of 11KT, 11OHT, and 11KA4 than non-CHC users (all p’s < 0.001). After exclusion of CHC users, sex differences attenuated and was no longer significant for 11KT.

**Conclusion:**

Females had lower concentrations of 11-OxyA than males, partly explained by use of CHC, as users had significantly lower concentrations of 11-OxyA than non-CHC users, except 11OHA4. These findings suggest an additional mechanism for CHC in treatment of hyperandrogenic conditions, in which 11-OxyA are elevated.

**Supplementary Information:**

The online version contains supplementary material available at 10.1007/s40618-025-02793-0.

## Introduction

Androgens and their precursors are produced in the gonads, the testes in males and the ovaries in females, as well as in the adrenal glands of both sexes [1]. Androgens play a crucial role in prenatal sexual development and differentiation of male gonads, the development of male characteristics, and sexual maturation during puberty in males [1, 2]. During puberty, the production of testosterone (T) and dihydrotestosterone (DHT), two central androgens, rises unevenly in males and females. A 20-fold increase in males leads to a nearly 15-fold difference in free T between the sexes [1–3]. A new piece in the puzzle of androgen effects in both sexes has emerged through a novel interest in adrenal androgens.

Traditionally, the adrenal gland has been associated with production of glucocorticoids, mineralocorticoids, and steroid hormone precursors such as dehydroepiandrosterone (DHEA). In the early 1950s, Dorfman and colleagues discovered and verified the existence of a novel androgen, the 11-oxygenated 11β-hydroxyandrostenedione (11OHA4) [4, 5], which was later controversially perceived as a regulatory end-product for T [6]. Today, however, 11OHA4 has been established to be a precursor for the 11-oxygenated androgens (11-OxyA) 11β-hydroxytestosterone (11OHT), 11-ketoandrostenedione (11KA4) and notably 11-ketotestosterone (11KT) and 11-ketodihydrotestosterone (11KDHT), where 11KT and 11KDHT act as endocrinologically active androgens that bind to the androgen receptor, with efficacy similar to T and DHT respectively [5, 7, 8]. Elevated concentrations of 11-OxyA are reported in a variety of androgen-related conditions like polycystic ovary syndrome (PCOS), congenital adrenal hyperplasia, castration-resistant prostate cancer, Cushing’s disease and premature adrenarche [9].

These 11-OxyA are synthesized through the 11-oxygenated pathway from T and androstenedione (A4) of the classical androgen pathway. A4 and T may be hydroxylated to 11OHA4 and 11OHT respectively, catalyzed by the 11β-hydroxylase (CYP11B1). These adrenal androgens may subsequently be synthesized to 11KA4 and 11KT respectively, catalyzed by the hydroxysteroid-11β-dehydrogenase 2 and reversely 1 (HSD11B2 and HSD11B1) [10]. Additionally, the A4 derived 11-OxyA may be converted to and from T derived 11-OxyA through 17β-HSD type 5 (AKR1C3) [5, 11]. Lastly, 11KT is a substrate for steroid 5α-reductase 1 (SRD5A1) which synthesizes 11KDHT [5, 8]. Although the adrenal gland has access to all enzymes catalyzing the 11-oxygenated pathway, there is evidence that the adrenal gland primarily secretes 11OHA4 into the bloodstream and only a negligible amount of other 11-OxyA [12, 13]. Subsequent conversions thus take place in peripheral tissues.

Several analytical challenges exist for the analyses of 11-OxyA, limiting the comparability between laboratories and studies [14]. Few studies have reported method-specific population-based references so far. In children, there was no sex differences in 11-OxyA [15], while in adults, sex differences were much smaller than for T, with males having slightly higher concentrations [16, 17]. Further, 11KA4 and 11KT were reported to decrease with age in males, while 11OHA4 and 11OHT increased in females [16]. Serum concentrations of all 11-OxyA in men, and 11KT and 11OHT in females, have been reported to correlate with BMI [16, 17].

In studies among healthy females, the concentrations of 11-OxyA did not differ with menstrual cycle phases [17, 18], and there were no associations with hormonal contraceptive use [17]. However, only 15 females used hormonal contraceptives, and the study did not separate between use of gestagen-only and combined hormonal contraceptives (CHC) containing both estrogen and gestagen [17]. Finally, 11-OxyA concentrations were reported to change across pregnancy, in which 11KT and 11OHT were lowered and 11KA4 and 11OHA4 were increased compared to non-pregnant females [19].

In the present study we aimed to describe the distribution of adrenal 11-OxyA (11OHA4, 11KA4, 11OHT, and 11KT) in a population-based young adult cohort of both sexes and explore the association with hormonal contraceptive use in females.

## Materials and methods

### Fit futures

The Fit Futures Study (FF) is a population-based longitudinal study following participants from adolescence into adulthood through three waves of data collection. In the first survey FF1 (2010–2011), all students in first grade of all upper secondary schools in Tromsø and Balsfjord municipalities were invited, and 1038 (93%) attended. Two years later, 868 students from the third year of the upper secondary schools attended Fit Futures 2 (2012–2013), including 132 not attending FF1. Nine years later in FF3 (2021–2022), all previous participants from FF1 and/or FF2 who were still alive were invited, and 705 (60%) attended. This cross-sectional study utilizes data only from FF3. The examinations took place at the Clinical Research Unit at the University Hospital of North Norway. FF3 was composed of questionnaires, clinical interviews, physical examinations and sampling of biological material including blood. All participants gave written consent before inclusion. The Regional Committee for Medical Health Research Ethics North Norway (REK Nord) approved the present study (Nr 479516).

#### Questionnaire

For this study we used self-reported data from the questionnaire regarding general information on age and sex assigned at birth, and specific information on pregnancy and contraceptive use. Information regarding diseases and medications were assessed in order to exclude participants with conditions or medication distinctly affecting the androgen concentrations, like pregnancy, leading to changes in 11-OxyA concentrations [19], or T substitution therapy.

Questions regarding contraceptive use included the specific types of contraceptives; contraceptive pill, implants, intrauterine devices, injections, transdermal, vaginal rings, and condom usage, classified by specific brand names and ATC-code. The contraceptives were categorized as CHC (estrogen-containing contraceptive pills, patches, and vaginal rings) or gestagen-only contraceptives (gestagen pills, intrauterine devices, implants or injections).

#### Measurements

Height was measured in centimeters with one decimal point and weight measured in kilograms to nearest hectogram in light clothing (Jenix DS 102 stadiometer). BMI was calculated (kg/m2).

### Laboratory analysis

Blood samples were obtained fasting between 08:00 and 12:00 (97.9% before 10:00), and sera were frozen within a short time frame, most within one hour and latest within two hours after sampling and temporarily stored at −30 °C after collection. Samples were monthly transferred to storage at −70 °C.

The analyses of 11-OxyA were conducted at the University Hospital of North Norway. Thawed sera were analyzed by LC-MS/MS, accompanied by in-house standard curves and quality controls to quantify concentrations of 11-OxyA, T and androstenedione. A detailed description of the laboratory methods has been published previously [20], and will be reviewed briefly here.

The analytes 11-ketotestosterone, 11KT; 11β-hydroxytestosterone, 11OHT; 11-ketoandrostenedione, 11KA4; and 11β-hydroxyandrostenedione, 11OHA4 were purchased from Steraloids Inc (Newport, Rhode Island, USA) to create calibration curves for each analyte. Additionally, isotope labelled 11KT-d3 was purchased from Cayman Chemical Group (Ann Arbor, Michigan, USA); and 11OHT-d4, 11KA4-d10 and 11OHA4-d4 from Cambridge Isotope Laboratories (Andover, Massachusetts, USA) to be used as internal standard. Last, for quality control (QC) of testosterone and androstenedione, the CE-IVD MassChrom^®^ Steroids panel 2 was purchased from Chromsystems Instruments & Chemicals (München, Germany). For the 11-OxyA, concentrations for low controls were spiked to 2 nM, and high controls were spiked to 20 nM.

LC-MS grade methanol and acetonitrile were purchased from Honeywell (Seelze, Germany), LC-MS grade formic acid, ammonium acetate and LC-grade tert-butyl methyl ether (TBME) was purchased from Merck KGaA (Dramstadt, Germany). Ultrapure water (18.2 MΩ) was obtained from a Millipore Integral 5 system (Molsheim, France).

A seven-point calibration curve was prepared in methanol: water (1:1) ranging from 0.025 nM to 25 nM for the 11-OxyA and from 0.13 nM to 130 nM for testosterone and androstenedione. The labelled analytes were mixed to 3 nM for 11-OxyA and 30 nM for testosterone and androstenedione, in ultrapure H_2_O.

Extraction was performed on a Tecan Fluent 1080 liquid handler. Aliquots of 70 µL (samples, calibration standards, and QC samples) were dispensed into a 96-well plate (Sarstedt), followed by the addition of 60 µL IS-mix and 100 µL of 0.1 M ZnSO4:methanol (1:1) for protein precipitation. After shaking at 1500 rpm for 2 min, 500 µL TBME was added, and the plate was shaken at 1450 rpm for 3 min to extract steroids. The plate was centrifuged at 1600 rpm for 4 min (Hettich Rotina 320R), and the upper organic phase was transferred to a 1 ml 96-well collection plate (Waters). The solvent was evaporated under a nitrogen stream at 40 °C, and samples were reconstituted in 60 µL of 70% methanol containing 0.1% formic acid.

Chromatographic separation was achieved on an Acquity Cortecs T3, 120Å, 1.6 μm 2.1 × 100 mm column (Waters) maintained at 50 °C and flow rate 0.3 mL/min. A gradient system of 5 mM ammonium acetate and 0.1% formic acid in water (phase A), and 5 mM ammonium acetate and 0.1% formic acid in methanol: acetonitrile in a 1:1 ratio (phase B) was used. The gradient started with 40% phase B, increasing to 70% in 8 min, and maintaining at 95% for 30 s before returning to starting conditions. The autosampler injection volume was 4 µL at 6 °C, with a flow-through-needle as injector. The needle was washed for 6 s with 90% methanol after injection. Retention time and MS/MS parameters are presented in supplementary **Table S1**.

Mass spectrometer data was captured on a Waters Xevo TQ-XS mass spectrometer (Waters, Manchester, UK) in ESI positive and negative mode with source temperature, 150 °C; capillary voltage, 1 kV; cone gas flow, 150 L/h; desolvation gas temperature, 550 °C; desolvation gas flow, 1000 L/h; nebulizer pressure, 7 Bar.

The method was validated and found to be linear from 0.25 to at least 25 nM (r2 > 0.995 for 11KT, 11KA4, 11OHT and 11OHA4 respectively). Lower limit of quantification (LLOQ) was found to be 0.025 nM. Between-day coefficients of variation (CVs) for all analytes were < 10% on three consecutive days. Intraday precision values were evaluated by assaying three samples (low, medium and high concentration) six times on the same day. The CVs for all analytes were < 6% for all three levels. Accuracy for recovery tests was between 92% and 106%. Instrument precision CVs were found to be < 3% for the analytes.

Serum luteinizing hormone (LH) and follicle-stimulating hormone (FSH) were analyzed utilizing the Cobas 6000 instrument (Roche Diagnostics, F. Hoffmann-La Roche Ltd, Basel, Switzerland). CV for both was < 5%.

The analysis of estradiol was conducted at Haukeland University Hospital, Norway, utilizing ultra high-performance liquid chromatography (UPLC) (Shimadzu Nexerea LC) with Acquity UPLC BEH Phenyl 1.7 μm, 2.1 × 50 mm column (Waters) and 0.2 μm in-line filter (Waters) at 60 °C. For the mobile phase, 0.1% ammonium hydroxide in water and pure methanol was utilized, starting from 30% methanol to 67.2% in 5.35 min, with a total runtime of 9.45 min. Mass spectrometer data was captured on QTRAP 6500+ (SCIEX) in negative electrospray ionization mode with: temperature, 500 °C; ion spray, −4300 V; curtain gas, 20 psi; collision gas, 12 psi; ion source gas nr.1, 50 psi; ion source gas nr.2, 70 psi; declustering potential, −110 V; entrance potential, −10 V [21].

### Statistical analysis and data management

The statistical analysis was performed utilizing IBM SPSS statistics version 28.0.1.1 (released 25th of May 2021). Predetermined exclusion criteria included missing data on adrenal androgens, participants not fasting, pregnancy, and use of testosterone supplements. Due to homogeneous ethnicity with > 95% participants of Caucasian origin in the study sample, this was not further assessed [22, 23]. Normality was visually assessed from histogram plots, from kurtosis and skewness of the data, and utilizing the Shapiro-Wilk and Kolmogorov-Smirnov tests.

Due to non-normal, right-skewed distribution of 11-OxyA concentrations, the distributions in males and females are presented with medians and interquartile ranges (IQR), 2.5th and 97.5th percentiles in addition to means and standard deviations (SDs). Owing to the large sample size, parametric independent t-tests and ANOVA were still chosen for comparing the groups [24]. Reference intervals were derived from non-parametric 2.5th and 97.5th percentiles without outlier exclusion.

As ANOVA revealed that concentrations of 11-OxyA were similar in users of gestagen-only contraceptives and participants who did not use hormonal contraceptives, this variable was further dichotomized to CHC users and non-CHC users.

Finally, to ensure that the differences between female CHC users and non-users were not confounded by BMI and age, we performed multiple linear regression analyses with CHC use as exposure, adjusting for BMI and age.

## Results

**Fig. 1:**
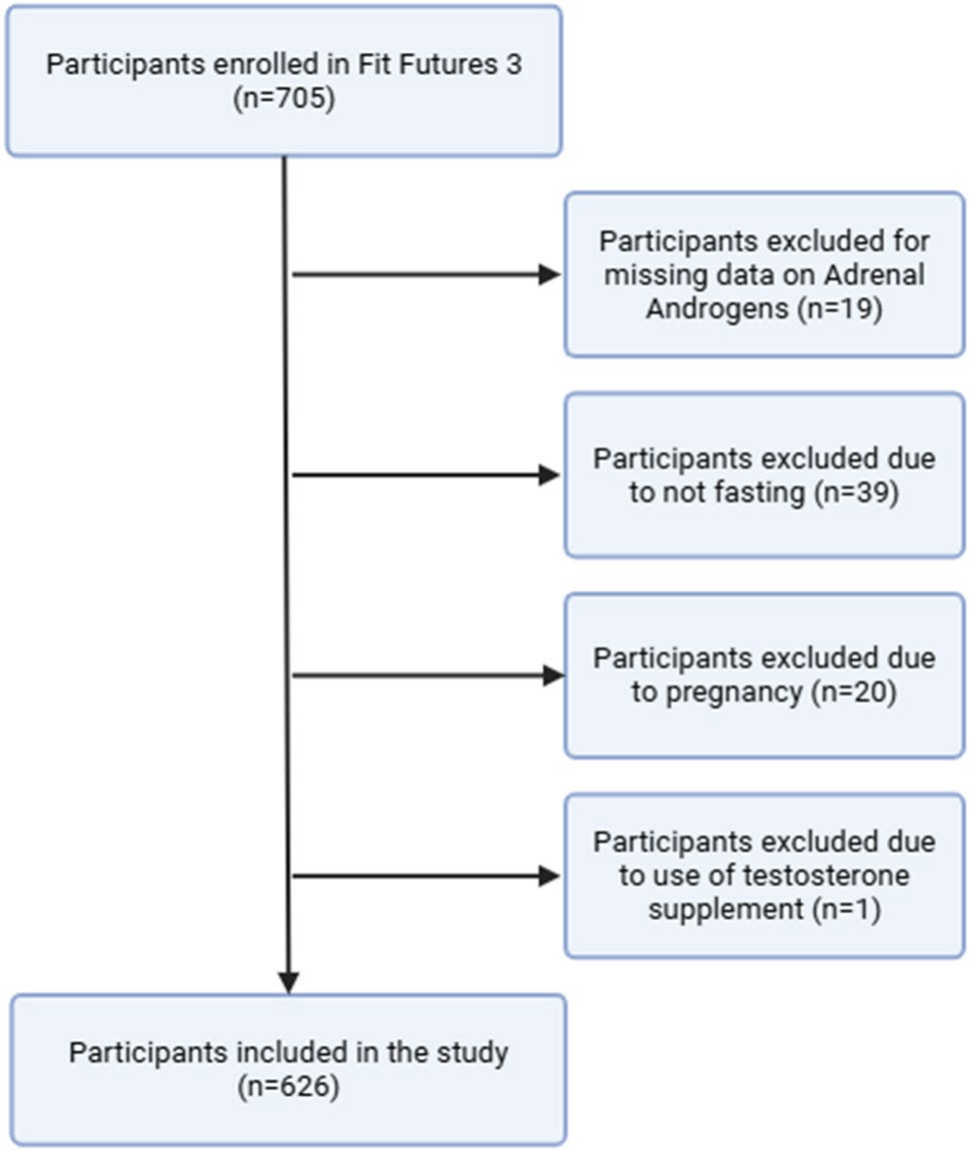
Flow diagram for inclusion and exclusion of the study

Of the initial 705 valid participants enrolled in Fit Futures 3, 79 participants were excluded based on predefined criteria, leaving a total of 626 participants in this study (Fig. 1).

Of these, 289 were males and 337 were females. Background characteristics are presented in Table 1. Participants were similar in age, ranging from age 26 to 35 years with a median of 27 years. Approximately one quarter of the females used CHC, one third used gestagen only contraceptives, and the remaining used no or non-hormonal contraceptives. LH, FSH, and estradiol were significantly lower in the CHC group compared to other groups, while T and androstenedione were lower in the gestagen-only group and even lower in the CHC group.

**Table 1:** Characteristics of the study participants, the Fit Futures Study, 2021-2022

	Male (*n*=289)		Female (*n*=337)	
Age (years)	26.9 (0.9)		26.9 (1.2)	
Height (cm)	179.3 (6.7)		166.4 (6.5)	
Weight (kg)	84.6 (16.5)		71.2 (15.0)	
BMI (kg/m^2^)	26.3 (4.7)		25.7 (5.4)	
Testosterone (nmol/L)	16.95 (5.43)		0.93 (0.44)	
Androstenedione (nmol/L)	3.48 (1.24)		3.93 (1.81)	
FSH (IU/L)	3.51 (2.37)		4.48 (2.66)	
LH (IU/L)	4.85 (1.85)		6.84 (7.16)	
Hormonal contraceptives (n%)				
CHC	n/a		86 (25.5%)	
Gestagen-only	n/a		124 (36.7%)	
None	n/a		127 (37.8%)	

Female hormonal contraceptive use	CHC (*n*=86)	Gestagen-only (*n*=124)		None (*n*=127)
BMI (kg/m^2^)	25.2 (5.1)	25.8 (5.4)		26.0 (5.6)
Testosterone (nmol/L)	0.83 (0.43)^a^	0.85 (0.33)^a^		1.07 (0.51)^b, c^
Androstenedione (nmol/L)	2.83 (1.35)^a, c^	3.92 (1.47)^a, b^		4.69 (2.01)^b, c^
Estradiol (pmol/L)	60.6 (153.0)^a, c^	289.0 (318.3)^b^		324.4 (290.1)^b^
FSH (IU/L)	2.10 (2.20)^a, c^	5.24 (2.25)^b^		5.35 (2.32)^b^
LH (IU/L)	2.37 (3.11)^a, c^	7.40 (5.49)^b^		9.32 (8.97)^b^
LH/FSH ratio	1.1 (0.7)^a^	1.5 (0.9)^a^		1.8 (1.2)^b, c^

BMI, Body mass index; CHC, Combined hormonal contraceptives; FSH, Follicle-stimulating hormone; LH, Luteinizing hormone; n/a, not available. Values are presented as means (SD), unless specified otherwise. For females, subsequent analysis of variance (ANOVA) for difference stratified by hormonal contraceptive use group was run. Statistical significance was determined as *p*<0.05, and marked as a, b, or c corresponding to Tukey’s honestly significant difference (HSD) post hoc test

^a^Significantly different from the “None” group

^b^Significantly different from the “CHC” group

^c^Significantly different from the “Gestagen-only” group

**Fig. 2:**
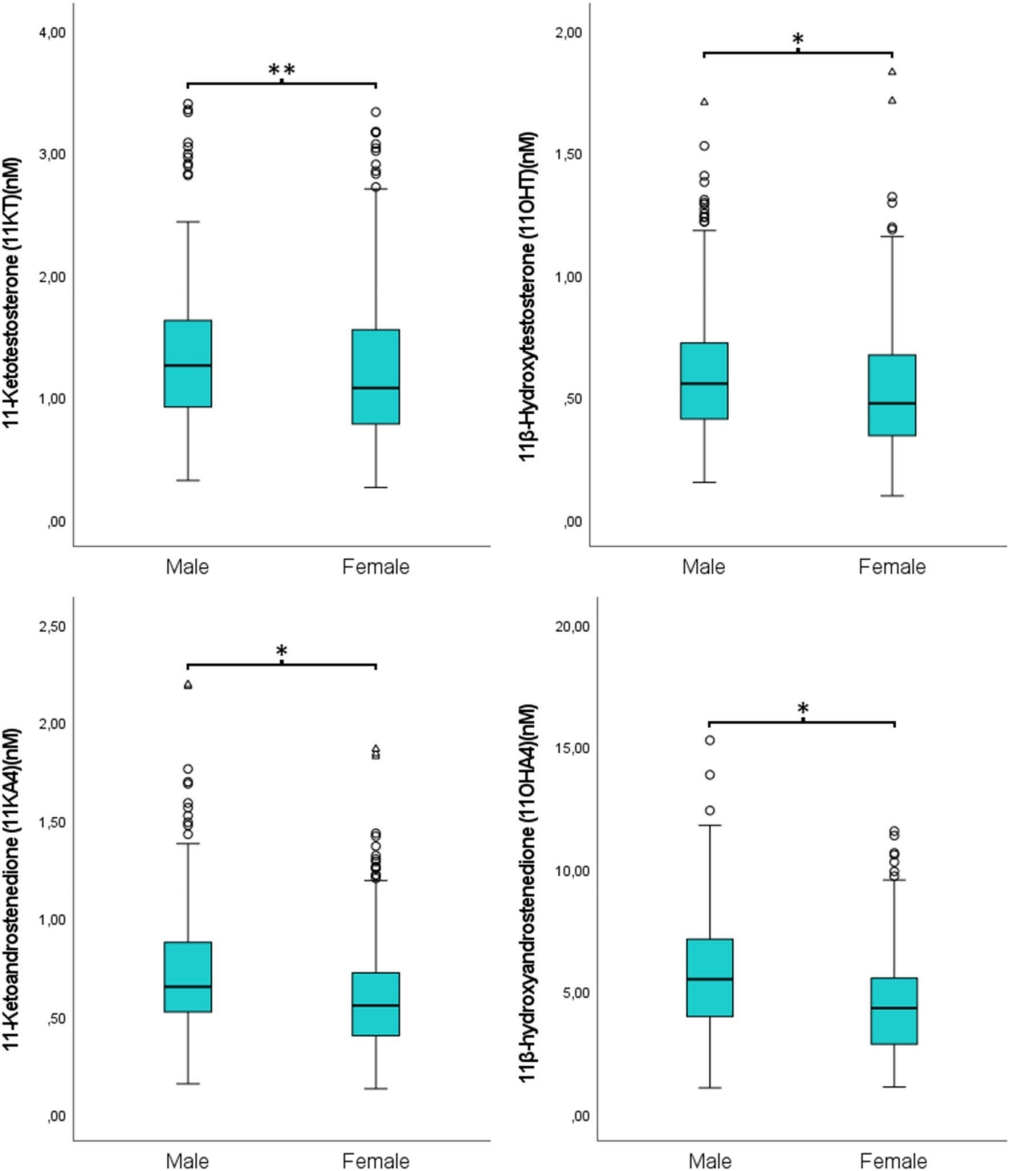
11-oxygenated androgens in males and females. Males (*n* = 289), females (*n* = 337).*p < 0.001,**p < 0.05

**Fig. 3:**
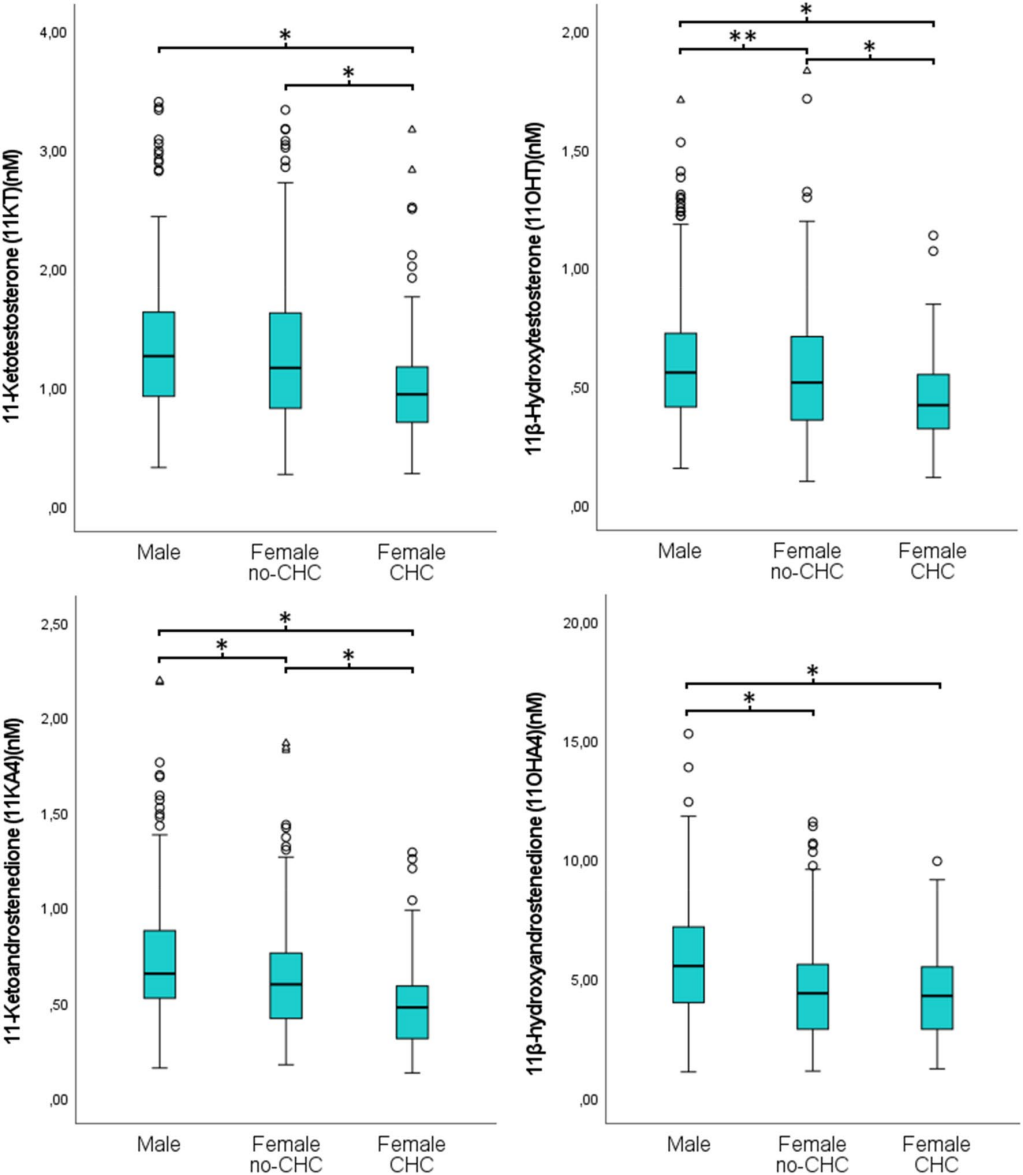
11-oxygenated androgens in females stratified by CHC use. CHC, Combined hormonal contraceptives. Males (*n* = 289), females non-CHC (*n* = 251), females CHC (*n* = 86). The females non-CHC group comprises both non contraceptive users and gestagen-only users, as these were combined. * - *p* < 0.001,** - *p* < 0.05

The distributions of the 11-OxyA in males and females are presented in Fig. 2, and for comparisons with previous studies in Table 2. Reference intervals for the four 11-OxyA investigated are presented in Table 3. Generally, males had higher concentrations of 11-OxyA compared to females, with 9.2% higher mean concentrations of 11KT, 15.2% of 11OHT, 23.1% 11KA4, and 30.1% 11OHA4 (all p’s < 0.01). Among females, users of CHC had significantly lower concentrations of 11KT, 11OHT and 11KA4, but not 11OHA4 (Table 4; Fig. 3). We found no statistically significant correlations between BMI and any of the 11-OxyA in either sex. In addition, linear regression analyses between CHC use and 11-OxyA adjusted for age and BMI, yielded results very similar to the unadjusted analyses (results not shown).

**Table 2:** 11-oxygenated androgens from the Fit Futures Study, 2021-2022, and previously published results from other cohorts

	Subject	11KT	11OHT	11KA4	11OHA4
*Data reported on healthy females*					
Current study – The Fit Futures Study	*n*=337 Age 26–35 (mean 27) Mean [SD] Median [IQR]	1.22 [0.60] 1.07 [0.78–1.55.78.55]	0.52 [0.26] 0.47 [0.34–0.67.34.67]	0.59 [0.28] 0.55 [0.40–0.72.40.72]	4.43 [1.98] 4.31 [2.84- 5.54]
Mody et al. 2025	*n*=78 Age median 31 Median [IQR]	0.71 [0.53–1.04.53.04]	0.37 [0.22–0.53.22.53]	0.49 [0.40–0.68.40.68]	3.89 [2.82–5.02.82.02]
Allaoui et al. 2024	*n*=75 Median [IQR]	T1 1.23 [0.87] T2 1.02 [0.62] T3 1.10 [0.79]	T1 0.43 [0.35] T2 0.44 [0.28] T3 0.44 [0.39]	T1 0.53 [0.46] T2 0.41 [0.30] T3 0.50 [0.37]	T1 3.83 [3.08] T2 3.63 [2.56] T3 4.06 [2.76]
Vitku et al. 2024	*n*=20 Median [IQR]	1.34 [1.08–1.66.08.66]	0.45 [0.33–0.48.33.48]	n/a	2.32 [1.37–3.00.37.00]
Schiffer et al. 2023	*n*=85 Age 21–49 (Median 30) Median [5^th^−95^th^%]	0.70 [<0.33–1.6.33.6]	0.39 [<0.33–0.78.33.78]	3.2 [1.6–5.4.6.4]	7.5 [3.4–14.0.4.0]
Caron et al. 2021	*n*=10 *n*=10 Age 20–40 Mean [SD]	Ft 0.85 [0.10] Ff 0.65 [0.10] Lt 0.87 [0.10] Lf 0.73 [0.10]	Ft 0.34 [0.04] Ff 0.31 [0.04] Lt 0.34 [0.04] Lf 0.30 [0.04]	Ft 0.81 [0.06] Ff 0.64 [0.06] Lt 0.83 [0.06] Lf 0.66 [0.06]	Ft 5.25 [0.39] Ff 3.43 [0.35] Lt 4.55 [0.40] Lf 3.17 [0.21]
Davio et al. 2020	*n*=72 Age 18–39 Median [IQR]	0.70 [0.53–1.06.53.06]	0.36 [0.23–0.52.23.52]	0.50 [0.40–0.70.40.70]	3.87 [2.85–5.00.85.00]
Nanba et al. 2019	*n*=100 Age 27–39 Median [IQR]	0.86 [0.63–1.26.63.26]	0.46 [0.30–0.75.30.75]	1.23 [0.87–1.83.87.83]	5.69 [3.91–8.64.91.64]
Skiba et al. 2019	*n*=163, Follicular *n*=184, Mid cycle *n*=241, Luteal Age 18–40 Median [min-max]	1.30 [0.33–7.61.33.61] 1.34 [0.03–5.79.03.79] 1.23 [0.09–4.57.09.57]	n/a	8.80 [0.07–28.84.07.84] 7.71 [1.10–31.67.10.67] 7.73 [0.57–27.97.57.97]	n/a
O’Reilly et al. 2017	*n*=49 Age 28 (IQR 23–32) Median [IQR]	1.5 [1.2–1.8.2.8]	0.2 [0.1–0.3.1.3]	2.7 [2.0–3.9.0.9]	6.8 [4.9–12.5.9.5]
*Data reported on healthy males*					
Current study – The Fit Futures study	*n*=289 Age 26–33(mean 27) Mean [SD] Median [IQR]	1.34 [0.56] 1.26 [0.92–1.63.92.63]	0.60 [0.27] 0.56 [0.41–0.73.41.73]	0.73 [0.31] 0.65 [0.52–0.88.52.88]	5.77 [2.49] 5.49 [3.94–7.15.94.15]
Allaoui et al. 2024	*n*=63 Median [IQR]	T1 1.13 [0.90] T2 0.92 [0.54] T3 1.03 [0.79]	T1 0.45 [0.45] T2 0.41 [0.27] T3 0.47 [0.32]	T1 0.53 [0.41] T2 0.36 [0.24] T3 0.57 [0.34]	T1 4.44 [2.80] T2 3.53 [2.80] T3 4.24 [2.69]
Schiffer et al. 2023	*n*=48 Age 22–47 (Median 34) Median [5^th^−95^th^%]	0.86 [<0.33–2.2.33.2]	0.47 [<0.33–1.1.33.1]	3.5 [1.42–6.2.42.2]	7.6 [2.9–14.0.9.0]
Turcu et al. 2021	*n*=10 Age 19–29 Median [IQR]	1.38 [1.35–1.41.35.41]	0.59 [0.55–0.62.55.62]	2.05 [1.97–2.14.97.14]	8.60 [8.22–8.99.22.99]
Davio et al. 2020	*n*=69 Age 18–39 Median [IQR]	0.99 [0.66–1.39.66.39]	0.43 [0.36–0.56.36.56]	0.57 [0.43–0.70.43.70]	4.10 [3.14–5.59.14.59]

11KT, 11-ketotestosterone; 11OHT, 11β-hydroxytestosterone; 11KA4, 11-ketoandrostenedione; 11OHA4, 11β-hydroxyandrostenedione; SD, standard deviation; IQR, interquartile range; n/a, not available; Ft, follicular total; Ff, follicular free; Lt, luteal total; Lf, luteal free; T1, The Tromsø study 3; T2, The Tromsø study 4; T3, The Tromsø study 5. Comparison of previously published 11OxyA concentrations. Some of the published data was not presented in nmol/L and therefore was converted to nmol/L for ease of comparison. Conversion from ng/dl to nmol/L was done by multiplying 0.0331 for 11KT and 11OHA4, 0.0328 for 11OHT, 0.0333 for 11KA4; and conversion from pg/ml to ng/dl by dividing by 10. Conversions were previously published by Davio et al. Lastly, results were rounded to the nearest two decimal points

**Table 3:** Distribution of 11-oxygenated androgens in males and females, the Fit Futures Study, 2021-2022

	Male (*n*=289)				Female (*n*=337)			
	Reference interval				Reference interval			
	Median	Mean (SD)	2.5%	97.5%	Median	Mean (SD)	2.5%	97.5%
11KT (nM)	1.257	1.335 (0.561)	0.514	2.948	1.074	1.222 (0.601)	0.393	2.878
11OHT (nM)	0.555	0.603 (0.265)	0.222	1.283	0.474	0.523 (0.255)	0.168	1.131
11KA4 (nM)	0.648	0.728 (0.312)	0.303	1.531	0.552	0.591 (0.278)	0.192	1.293
11OHA4 (nM)	5.488	5.765 (2.494)	2.028	11.412	4.311	4.431 (1.978)	1.504	9.423

11KT, 11-ketotestosterone; 11OHT, 11β-hydroxytestosterone; 11KA4, 11-ketoandrostenedione; 11OHA4, 11β-hydroxyandrostenedione. Reference intervals displayed as 2.5% and 97.5% percentiles

**Table 4:** Differences in 11-oxygenated androgens concentrations by use of combined hormonal contraceptives in females, the Fit Futures Study, 2021-2022

	Combined Hormonal Contraceptives		
	Yes (*n*=86)	No (*n*=251)	*P* for difference
11KT (nM)	1.03 (0.54)	1.29 (0.61)	<0.001
11OHT (nM)	0.44 (0.20)	0.55 (0.27)	<0.001
11KA4 (nM)	0.49 (0.24)	0.63 (0.28)	<0.001
11OHA4 (nM)	4.41 (1.99)	4.44 (1.96)	0.450

11KT, 11-ketotestosterone; 11OHT, 11β-hydroxytestosterone; 11KA4, 11-ketoandrostenedione; 11OHA4, 11β-hydroxyandrostenedione. Values are displayed as mean (SD). P-value derived from independent t-test

When CHC users were excluded from the analyses, the sex differences were attenuated, and no longer significant for 11KT (Fig. 3). As most CHC use was through oral administration, and only 10 participants (11% of CHC users) used transdermal or vaginal formulations, subgroup analyses stratified by route of administration were not feasible. The active estradiol substance was ethinylestradiol in all CHC formulations used, and thus stratified analyses based on type of estrogen analogues were not performed.

## Discussion

This study presents the distribution of 11-OxyA from a population-based sample of young adults, with a special emphasis on contraceptive use in females. Although females in general had lower concentrations of all 11-OxyA, these differences were partly explained by use of CHC, as CHC use was associated with lower concentrations of 11-OxyA.

This is one of the largest studies among adults to report the distribution of 11-OxyA in both sexes. When comparing the concentrations revealed in this study with other population-based studies, there are considerable discrepancies between them. For females, the mean or median 11KT concentrations have been reported from 18% lower to 40% higher than in the present study [16–18, 20, 25–28]. Even larger discrepancies have been reported for 11KA4 concentrations, ranging from similar concentrations [16, 20, 29] to some reporting 5-fold [17] and 13-fold higher concentrations than in our population [18]. The discrepancy between the studies may relate to differences in populations included, and as shown in this study, for females also information regarding use of CHC. In addition, there is also a concern regarding the lack of standardization of laboratory methods. LC-MS/MS assay is widely used for the analyses of 11-OxyA, but to our knowledge, there is no external control assessment program for these analytes, and no studies have directly compared results from different laboratories [14]. Recently, artificial elevations of 11OHA4 and 11KA4 in LC-MS/MS assays due to temperature-dependent non-enzymatic conversion from cortisol and cortisone respectively were reported [30]. Also, longer periods of time from sampling to processing in room temperature has been shown to increase the concentration of 11KT through synthesis from 11KA4 mediated by mononuclear cells, such as the natural killer cell [31]. Lastly, the 11-OxyA have been shown to exhibit a circadian rhythm, falling in concentration during the day and night before rising again before 8 am [32]. In the present study, the risk of these biases was minimized by blood sampling in the morning with further centrifugation and freezing within a short time frame. Further, the use of an automated liquid handling robot minimized the time spent in room temperature during preparation and the risk of spontaneous conversion. The increasing focus on the role of 11-OxyA in different clinical conditions will actualize the need of standardization of these analyses.

Sex differences in 11-OxyA concentrations have been established prior to this study. Generally, it seems that there are differences in 11-OxyA concentration between the sexes [16], albeit much smaller differences than for other androgens such as T, DHEA and its sulfate DHEAS [33–36]. In a study by Davio and colleagues, they reported a 1.1-fold higher concentration of all 11-OxyA investigated in males [16]. Other studies have also reported concentrations on 11-OxyA in young adults without investigating sex differences [17, 20], and as shown in Table 2, the concentrations of 11-OxyA were higher in males than in females in most studies reporting from both sexes. On the other hand, Caron and colleagues have reported higher concentrations of 11-OxyA in females [25]. Again, differences regarding populations, prevalence of contraceptive use and laboratory challenges may affect the results.

We did not find any correlation between 11-OxyA and BMI in either sex. Previous studies have reported inconsistent results, where some studies reported associations between 11-OxyA and BMI [16, 17, 37], while other reported no associations or associations with only some 11-OxyA [18, 27, 38, 39]. These contrasting findings may be due to aforementioned population differences. Quinkler and colleagues found AKR1C3 activity and conversion of androstenedione to testosterone mediated by AKR1C3 associated with BMI in subcutaneous tissue and the inverse in omental tissue [40]. Interestingly, they also found AKR1C3 expression to decrease with patient weight loss.

This study is the first of sufficient power examining the impact of hormonal contraceptives on 11-OxyA. A recent study by Schiffer and colleagues [17] reported no differences in serum concentrations of 11-OxyA between female oral hormonal contraceptive users and non-users. However, the study included only 15 users, and both CHC and gestagen-only contraceptives were included in the user group. In contrast, we found significantly lower serum concentrations of 11KT, 11OHT and 11KA4 in females using CHC, whereas users of gestagen-only contraceptives had similar concentrations of 11-OxyA as non-users. In addition, the sex differences in 11-OxyA concentrations were attenuated after exclusion of CHC users, and no longer significant for 11KT.

CHC are used as first-line treatment for PCOS [41], a common condition among females characterized by combinations of clinical or biochemical hyperandrogenism, menstrual disturbances, and polycystic ovary morphology. By inducing production of Sex Hormone Binding Globulin (SHBG) from the liver, peroral CHC reduces the fraction of T available for the androgen receptor, and thereby the symptoms. Further, the negative feedback from the CHC on the pituitary leads to a reduction of luteinizing hormone (LH), and a consecutive reduced production of T from the ovaries. Thus, both total and free T concentrations have been reported lowered by the use of CHC [42]. Additionally, CHC has been reported to reduce levels of androstenedione and endogenous estradiol in serum [43, 44]. In accordance, lower LH, testosterone, androstenedione and endogenous estradiol was also seen in CHC users in the present study. Another known effect of peroral CHC use is an increase in cortisol binding globulin, which leads to a temporarily lowered free cortisol concentration, followed by feedback regulation to increase the total cortisol concentration and reinstate the original free cortisol concentration [42, 45]. Although the mechanism for this is not yet fully known, it has been suggested that this may eventually lead to lowered adrenocorticotropin hormone (ACTH) secretion, supported by studies showing that both DHEA and DHEAS are lowered by CHC use [34–36]. Several studies have shown that PCOS patients also have elevated concentrations of 11-OxyA [27, 38, 46–48], and our results thus suggest another mechanism for the beneficial effects of CHC in PCOS.

Further studies are needed to understand the underlying mechanisms behind the lower 11-OxyA concentrations in CHC users. As the ovaries do not produce 11-OxyA [49], it is unlikely that the mechanism is by diminished LH-secretion. Furthermore, it is still not known whether 11-OxyA have affinity to and bind SHBG. While CHC use does increase serum SHBG, we utilized the LC-MS/MS technique which measures the total 11-OxyA concentration in serum, implying that SHBG binding could not explain the lower total concentration of 11-OxyA in CHC users. As previously mentioned, the primary secreted 11-OxyA is 11OHA4 [12], which was the only investigated 11-OxyA where serum concentrations were unaffected by CHC use in our study, eliminating the adrenals as the target tissue. Thus, lowered concentrations of 11-OxyA by potentially lowered ACTH stimulus in CHC users is unlikely, even though ACTH was not measured in the present study. Conversely, this suggests a currently unknown mechanism where CHC use affects 11-OxyA synthesis in peripheral tissues.

There are several strengths to our study. First, we included a quite homogenous population-based sample of young healthy adult participants, with high retention from previous Fit Future studies. This provides population-based reference ranges for young Caucasian adults. Second, the study included exhaustive questionnaires providing data on health and lifestyle including detailed information regarding contraceptive use. Lastly, 11-OxyA measurements were conducted by LC-MS/MS assay with favorable pre-analytic conditions concerning pre-analytic pitfalls.

Conversely, there are also clear limitations with our study. Cross-sectional studies may only give a snapshot view of participants’ health and cannot be inferred as causal associations. As the study sample is limited in representation across age groups and ethnicities, the data may not be valid for other age groups and ethnicities. Finally, as there is no external control program for 11-OxyA available, comparisons between different laboratories are challenging.

## Conclusion

We have described the distribution of the 11-OxyA 11KT, 11OHT, 11KA4 and 11OHA4 in healthy young Caucasian adults and based on our validated method propose a reference range from the 2.5% percentile to the 97.5% percentile. Females had slightly lower concentrations of 11-OxyA than males. Among females, CHC users had lower concentrations of 11-OxyA than non-users with the exception of 11OHA4. Based on our findings, we hypothesize an additional mechanism for CHC in the treatment of hyperandrogenic conditions like PCOS, in which 11-OxyA are elevated.

## Supplementary Information

Below is the link to the electronic supplementary material.

Supplementary Material 3
