# Supplementary material for: 11-oxygenated androgens in healthy young adults: Does use of hormonal contraceptives matter? The Fit Futures Study
Source: J Endocrinol Invest. 2026 Feb 6;49(5):1059–70. doi: 10.1007/s40618-025-02793-0 (PMC13083356; doi:10.1007/s40618-025-02793-0)
Supplement: Supplementary file 1 — Supplementary Material 3 [file 40618_2025_2793_MOESM1_ESM.pdf]

Supplemental material

11-oxygenated androgens in healthy young adults. Does use of hormonal contraceptives matter? The Fit Futures Study.

Torkild Pettersen[1], Ole-Martin Fuskevåg[1,2], Yngve A. Figenschau[1,3], Elin K. Evensen[4], Anne-Sofie Furberg[5,6], Anne Winther[7], Guri Grimnes[1,8]

Affiliations

1. Tromsø Endocrine Research Group, Department of Clinical Medicine, UiT The Arctic University of Norway, Tromsø, Norway.
2. Department of Laboratory Medicine, University Hospital of North Norway, Tromsø, Norway.
3. Diagnostic Clinic, University Hospital of North Norway, Tromsø, Norway.
4. Department of Health and Care Sciences, UiT The Arctic University of Norway, Tromsø, Norway.
5. Department of Microbiology and Infection Control, University Hospital of North Norway, Tromsø, Norway.
6. Faculty of Health and Social Sciences, Molde University College, Molde, Norway.
7. Division of Internal Medicine, University Hospital of North Norway, Tromsø, Norway.
8. Division of Medicine, University Hospital of North Norway, Tromsø, Norway.

Correspondence:

Torkild Pettersen, Department of Clinical Medicine, UiT The Arctic University of Norway, Tromsø, Norway.

E-mail: Torkild.pettersen@uit.no

Table S1. Retention time and MS/MS parameters

Analyte	RT (min)	Quantifier MRM transition (m/z)	Qualifier MRM transitions (m/z)	Cone (V)	Collision (eV)
11-Ketotestosterone	3.89	303.2>121	303.2>259	40	23/23
11-Ketotestosterone-d3	3.86	306.2>121	306.2>262		23/23
11-Hydroxytestosterone	4.60	305.2>121	305.2>287	40	17/17
11-Hydroxytestosterone-d4	4.56	309.2>121	309.2>291		17/17
11-Ketoandrostenedione	3.46	301.2>257	301.2>121	40	23/23
11-Ketoandrostenedione-d10	3.41	311.2>125	311.2>265		23/23
11-Hydroxyandrostenedione	4.22	303.2>267	303.2>285	40	18/18
11-Hydroxyandrostenedione-d4	4.18	307.2>270	307.2>289		21/21
Testosterone	6.31	289.1>97	289.1>109	40	24/24
Testosterone-d3	6.26	292.1>97	292.1>109		24/24
Androstenedione	5.78	287.1>109	287.1>97	40	23/23
Androstenedione-d3	5.73	290.1>109	290.1>100		23/23

Table S1: MRM, Multiple reaction monitoring; m/z, Mass-to-charge ratio; V, Voltage; eV, Electronvolt.
